# The Role of *Staphylococcus aureus* Virulence Factors in Skin Infection and Their Potential as Vaccine Antigens

**DOI:** 10.3390/pathogens5010022

**Published:** 2016-02-17

**Authors:** Keenan A. Lacey, Joan A. Geoghegan, Rachel M. McLoughlin

**Affiliations:** 1 Host Pathogen Interactions Group, School of Biochemistry and Immunology, Trinity Biomedical Sciences Institute, Trinity College Dublin, Dublin 2, Ireland; klacey@tcd.ie; 2 Microbiology Department, Moyne Institute of Preventive Medicine, Trinity College Dublin, Dublin 2, Ireland; geoghegj@tcd.ie

**Keywords:** *Staphylococcus aureus*, skin infection, virulence factors, cell wall-anchored proteins, vaccine development

## Abstract

*Staphylococcus aureus* (*S. aureus*) causes the vast majority of skin and soft tissue infections (SSTIs) in humans. *S. aureus* has become increasingly resistant to antibiotics and there is an urgent need for new strategies to tackle *S. aureus* infections. Vaccines offer a potential solution to this epidemic of antimicrobial resistance. However, the development of next generation efficacious anti-*S. aureus* vaccines necessitates a greater understanding of the protective immune response against *S. aureus* infection. In particular, it will be important to ascertain if distinct immune mechanisms are required to confer protection at distinct anatomical sites. Recent discoveries have highlighted that interleukin-17-producing T cells play a particularly important role in the immune response to *S. aureus* skin infection and suggest that vaccine strategies to specifically target these types of T cells may be beneficial in the treatment of *S. aureus* SSTIs. *S. aureus* expresses a large number of cell wall-anchored (CWA) proteins, which are covalently attached to the cell wall peptidoglycan. The virulence potential of many CWA proteins has been demonstrated in infection models; however, there is a paucity of information regarding their roles during SSTIs. In this review, we highlight potential candidate antigens for vaccines targeted at protection against SSTIs.

## 1. *Staphylococcus aureus* Skin Infection

*Staphylococcus aureus* (*S. aureus*) is one of the leading causes of skin and soft tissue infections (SSTIs). SSTIs caused by *S. aureus* account for over 10 million outpatient visits and almost 500,000 hospital admissions in the United States each year [1]. Treatment of these infections is significantly hampered by the pathogen’s propensity to acquire antibiotic resistance. In particular, community-acquired methicillin resistant *S. aureus* (CA-MRSA) skin infections are occurring with increasing frequency in healthy individuals with no identified healthcare-associated risk factors [2]. It is estimated that 90% of CA-MRSA infections present as SSTIs [3]. Over the past 10 years, MRSA has become resistant to even last resort antibiotics. Vaccines targeted against *S. aureus* may offer a potential solution to this raging epidemic of antimicrobial resistance; however, despite significant efforts, an effective vaccine remains elusive. *S.* *aureus* vaccine development has been hampered by a fundamental lack of understanding of the correlates of immune protection in humans, and there is limited knowledge of which elements of the immune response are important in recovery from or prevention against *S. aureus* infection. In particular, there is a paucity of information on whether or not distinct immune mechanisms are important in providing site-specific protection against *S. aureus* infection.

## 2. Requirements for an Effective Anti-*S. aureus* Vaccine

A number of *S. aureus* vaccines have reached clinical trials, all of which were composed of a single target antigen that was designed to induce neutralising and opsonising antibodies [4], and although these vaccines produced robust humoral immunity, and proved efficacious in pre-clinical models, they did not prevent or attenuate infection in clinical trials [5]. The failure of these immunization strategies to confer protection in humans would suggest that antibodies alone are not sufficient to provide protection against *S. aureus*. This is somewhat unsurprising, as B cell deficiencies in humans and mice do not result in an increased frequency or severity of *S. aureus* disease [5,6,7]. There is increasing evidence that T cells, in particular T helper (Th) cells, have an important role in the immune response to *S. aureus* [8]. It is now widely accepted that protection against *S. aureus* requires a robust T cell response, in particular Th1 and Th17 cells, which are critical for controlling phagocytic cell responses and thus facilitating bacterial clearance [4,8]. Interestingly, it has been shown that model vaccines can actually confer protection against *S. aureus* infection in the complete absence of antibodies provided there is a robust T cell response [7,9]. Patients suffering from a range of conditions that affect Th cells are at greater risk of developing *S. aureus* infections [6,10,11,12]. Autosomal dominant hyper-IgE syndrome (AD-HIES) patients are susceptible to recurrent staphylococcal skin and lung abscesses [11]. AD-HIES results in impaired Th17 cell development, however, CD4^+^ T cells retain the ability to differentiate into other subsets of Th cells [13]. Interestingly, these patients are not more susceptible to *S. aureus* bloodstream infection, suggesting that Th17 responses are particularly important during skin and respiratory site infections, but may be less important during systemic infection. Similarly, mice deficient in interleukin-17A (IL-17A) and IL-17F showed no difference in pathology compared to the wild type strain following systemic infection, however, they had increased susceptibility to mucocutaneous *S. aureus* infections [14]. In contrast, a recent study reports that Th1 cells play a crucial role in protection against systemic *S. aureus* infection in mice and are expanded in the circulating blood of patients recovering from *S. aureus* bloodstream infection [15]. These studies highlight the concept that specific immune responses may be of greater importance at distinct sites of infection. Consequently the requirements for a vaccine may need to be tailored to the type of infection that it is aimed at preventing. A universal anti-*S. aureus* vaccine may never be realised [16] and instead, a vaccine targeting specific clinical manifestations may need to be pursued. As skin is the most frequent site of *S. aureus* infection [17], a vaccine specifically against SSTIs would be of great benefit. If prophylactic vaccines or other forms of immunotherapy to treat *S. aureus* SSTIs are to be developed as an alternative to antibiotics, we need a greater understanding of the specific roles of individual virulence factors during infection at this site, as they may be important targets in future therapies.

## 3. Skin

### 3.1. Skin Structure

**Figure 1:**
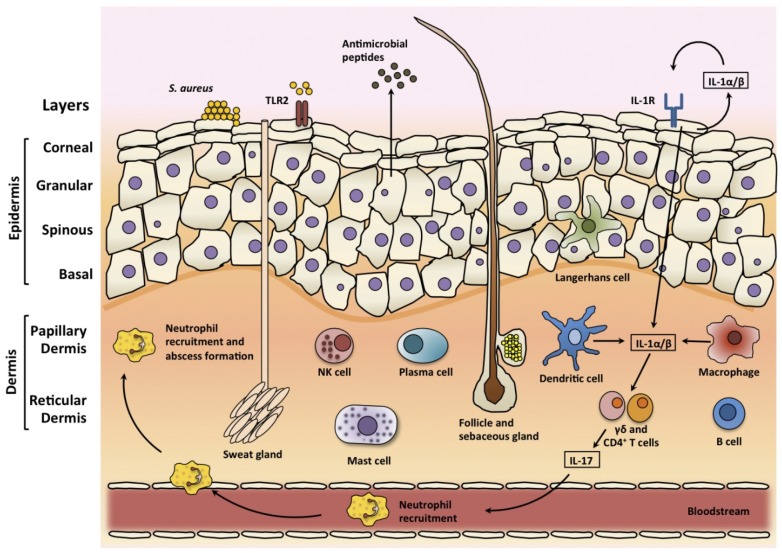
The architecture and immune responses of the skin. The epidermis is composed of layers of keratinocytes, including the corneal, granular, spinous, and basal layers. Sweat glands, sebaceous glands, and hair follicles span these layers. There are resident skin immune cells including Langerhans cells in the epidermis and dendritic cells, macrophages, mast cells, B and T cells, plasma cells and natural killer (NK) cells in the dermis. During *S. aureus* skin infection, these cells produce pro-inflammatory cytokines, chemokines and adhesion molecules, which can promote the recruitment of neutrophils from the bloodstream. Pro-inflammatory cytokines also induce the production of antimicrobial peptides that have bacteriostatic or bactericidal activity against *S. aureus.* Toll-like receptor 2 (TLR-2) is activated by *S. aureus* lipoproteins and lipoteichoic acid, and interleukin-1 receptor (IL-1R) is activated by IL-1α and IL-1β. IL-1 promotes the production of IL-17 and related cytokines from T cells. Through the production of IL-17, γδ and CD4^+^ T cells induce the expression of neutrophil-attracting chemokines and granulopoiesis factors thus promoting neutrophil recruitment and abscess formation.

The skin is the largest organ of the body and with the underlying fat layers, fascia, and muscle, represents the majority of the tissue in the body [18]. The corneal layer is the outermost layer of the skin and it is composed of terminally differentiated keratinocytes which have highly cross-linked keratin fibrils (Figure 1) [19]. The granular, spinous and basal layers lie below the corneal layer, and together with the corneal layer they make up the epidermis. Keratinocytes migrate from the basal layer up to the corneal layer from where they are eventually shed. This corneal layer provides the physical barrier of skin and it consists of dead keratinocytes, which are devoid of organelles [19]. This process of migration occurs constantly, allowing the epidermis to be continuously renewed. Beneath the epidermis resides the dermis layer, which is subdivided into the papillary dermis and the reticular dermis. The dermis is mainly composed of collagen and elastin fibres, but there are a number of skin structures that span the layers, such as hair follicles, sweat glands and sebaceous glands [18].

Although the skin is an important physical barrier to the invasion of pathogens, it is colonised with a normal microbiome; the species of which vary depending on the location, and other factors such as the temperature, pH, the presence of moisture, sebum, salt and fatty acids. The normal flora includes various species of staphylococci, propionibacteria, corynebacteria and yeasts. It is thought that the normal flora acts as a competitive inhibitor of non-commensal pathogenic microbes [19]. Some skin colonising microorganisms, in particular *S. aureus* and β-haemolytic group A streptococci but also Gram-negative bacteria, viruses and fungi, have the potential to cause infection, particularly when the skin barrier is breached [20].

### 3.2. Skin Infections

SSTIs are caused by microbial invasion of the layers of skin and underlying soft tissues. SSTIs have variable clinical presentations, etiology and severity. Infections may occur at sites where the skin barrier has been breached, such as a wound or surgical site infection. However, infections may also appear without apparent breach of the skin barrier, such as folliculitis occurring at hair follicles, or furuncles and carbuncles forming at pores [21]. The involvement of deeper layers such as the dermis and/or subcutaneous tissues leads to cellulitis [21], with the involvement of yet deeper tissues, such as underlying muscle leading to fasciitis. SSTIs are common and can affect all age groups, however, certain conditions such as trauma, immunosuppression, certain skin conditions and drug use may predispose an individual to SSTIs. *S. aureus* is capable of causing infections at all mentioned sites in the skin and in some instances outbreaks of *S. aureus* SSTIs can occur. These are mainly seen in cases where there is close body contact, in groups such as prisoners, athletes and soldiers [3]. CA-MRSA strains from the lineage USA300 are the most common cause of skin infections and 97% of all MRSA SSTI cases were caused by this lineage [22]. Many *S. aureus* SSTIs are self-limiting, however, complicated SSTIs can occur, and this often leads to the formation of a large abscess [23]. Abscesses can form in the dermis, epidermis and subcutaneous tissue and function primarily to contain the pathogen, preventing the spread of infection to adjacent healthy tissue [24]. Although abscess formation is part of the body’s defence mechanism, they can cause significant pathology and lead to benign or malignant obstruction in tissues. They can rupture, releasing bacteria into the surrounding tissue and local inflammation at the site of the abscess can lead to painful swelling for the patient [25]. An abscess begins as an acute localised inflammatory response to the invading bacteria [24]. The abscess forms and becomes a collection of pus composed of live and necrotic neutrophils, tissue debris and live bacteria, encased in a fibrous capsule [24]. Severe SSTIs may also lead to dermonecrosis of adjacent skin tissue.

### 3.3. Skin Immune Response

The skin possesses a robust innate and adaptive immune system to tackle infection if the skin barrier is breached (Figure 1). There are many resident immune cells in the skin including specialised dendritic cells known as Langerhans cells which are resident in the epidermis [19]. In the dermis, there are dendritic cells, macrophages, mast cells, T and B cells, plasma cells, and natural killer (NK) cells and their contribution to the cutaneous immune response has been the subject of a number of previous reviews [19,26]. Keratinocytes are very important in the cutaneous immune response. They produce large quantities of IL-1α, tumour-necrosis factor α (TNFα) and antimicrobial peptides such as β-defensins in response to various stimuli, including the presence of bacteria [26]. Keratinocytes also produce a large number of chemokines and other immunoregulatory cytokines in response to stimulation. These products in turn activate resident innate immune cells in the skin, further up-regulating expression of other inducible mediators and facilitating the recruitment of additional immune cells from the blood (Figure 1) [26]. Keratinocytes recognise the presence of *S. aureus* using pattern recognition receptors. Toll-like receptor 2 (TLR-2) on keratinocytes recognise pathogen associated molecular patterns (PAMPs) including peptidoglycan and lipopeptides [27]. Keratinocytes also express IL-1 receptor (IL-1R), which is activated by both autocrine and paracrine produced IL-1α and IL-1β [17]. Both IL-1R and TLR-2 signal through myeloid differentiation primary-response protein 88 (MyD88) to activate downstream signalling, which induces the nuclear factor-κB (NF- κB) and mitogen-activating protein kinase (MAPK) signalling pathways, leading to the transcription of proinflammatory genes, such as TNFα and IL-6, which recruit neutrophils during *S. aureus* skin infections [28,29]. A skin infection model using MyD88-deficient mice found that these mice had a reduced capacity for neutrophil recruitment and cytokine production, resulting in increased susceptibility to infection. Further study identified the IL-1R-MyD88 signalling axis as being primarily important for recruitment of neutrophils and protection against *S. aureus* SSTIs [30].

Clinical studies have highlighted the importance of the IL-1–IL-17 axis in cutaneous host defence against *S. aureus* [17]. Patients with IL-1 receptor-associated kinase 4 (IRAK4) deficiency, where IL-1R and TLR signalling is impaired, and AD-HIES patients who have defective IL-17 responses, were found to suffer from recurrent *S. aureus* SSTIs [13,28,31]. IL-1 functions to promote the production of IL-17 and related cytokines from Th17 cells but also from subpopulations of γδ T cells [32]. These IL-17 producing cells, comprising both Th17 cell and γδ T cells, have an important role in epithelial site immune responses. Through the production of IL-17A and IL-17F, they induce the expression of neutrophil-attracting chemokines and granulopoiesis factors thus promoting neutrophil recruitment and abscess formation [33,34,35]. In addition, IL-17 can also induce keratinocytes to produce antimicrobial peptides [36]. Murine models have shown that the related Th17 cytokine IL-22 may also be involved in protection against SSTIs [35,37]. In a murine skin abscess model, the inhibition of either IL-17A or IL-22 alone resulted in significantly larger lesion size, indicating that both IL-17A and IL-22 are necessary for local control of SSTIs [38]. A recent study demonstrated that a model vaccine that specifically drove Th17 (IL-17 and IL-22) immunity was able to contain *S. aureus* SSTIs [37]. Immunization with the model vaccine resulted in reduced bacterial burden in the abscess, in addition to a reduction in abscess volume and the level of dermonecrosis.

## 4. Virulence Factors Involved in the Pathogenesis of *S. aureus* SSTIs

### 4.1. Membrane Damaging Toxins

Numerous secreted virulence factors are produced by *S. aureus*, including a number of membrane damaging toxins capable of forming pores in the cytoplasmic membrane of host cells leading to cell lysis.

Bi-component leukocidins form an octameric structure of alternating S and F subunits that form a β-barrel pore spanning the lipid bi-layer of the host cytoplasmic membrane resulting in lysis [39]. Panton Valentine Leukocidin (PVL) is a β-barrel pore-forming cytotoxin that binds to the complement receptors C5aR and C5L2 on the surface of neutrophils [39]. Human and rabbit neutrophils are susceptible to the cytolytic activity of PVL; however, PVL cannot efficiently recognise the C5aR receptor on murine cells making them resistant [39,40]. LukAB and LukED are bi-component leukocidins that are closely related to PVL but recognise different host cell receptors. Although purified LukED, LukAD and PVL induce inflammation in rabbit skin [41,42], animal models have failed to demonstrate a clear role for bi-component leukocidins in the pathogenesis of skin infection [43,44,45]. There was little difference in infection outcome when *S. aureus* strain LAC and an isogenic PVL-deficient mutant were compared in rabbit skin abscess models. Several studies have demonstrated increased virulence of PVL-deficient mutants in murine models of skin abscess formation. However, these results must now be interpreted in light of the recent discovery that leukocidins antagonise each other’s activity by forming inactive hybrid complexes that compete for receptors [46]. PVL-deficient mutants, for example, display increased LukED activity *in vivo* [46]. Further studies are needed to fully understand the role of bi-component leukocidins in SSTI.

The role of the heptameric β-barrel forming alpha toxin (Hla) *in vivo* is much better understood. The Hla monomer binds to host cells and assembles as a heptamer forming a lipid bi-layer spanning β-barrel pore in a similar manner to the bi-component leukocidins. Hla has activity toward a variety of host cell types, including human keratinocytes, epithelial cells, lymphocytes and erythrocytes although it is unable to lyse neutrophils [47,48,49,50]. The receptor for Hla is A Disintegrin And Metalloprotease 10 (ADAM10) [51], which is expressed on leukocytes but also widely expressed by many cells in the liver, heart, kidney, lung, and in lymphoid tissues [52]. The Hla-ADAM10 interaction leads to the disruption of host cell membranes, and during skin infection this contributes to the necrosis of the epidermis and dermis layers [51,53]. Hla contributes significantly to the severity of USA300 skin infections. Upon subcutaneous infection, there was a striking difference in lesion phenotype between the wild type and Hla-deficient LAC infected mice [53]. Mice challenged with a Hla-deficient mutant of LAC formed significantly smaller lesions with visibly less dermonecrosis than those infected with the wild type strain. In a rabbit infection model, the Hla-deficient mutant of LAC formed smaller lesions with lower bacterial burden in the abscess compared to wild type LAC [43]. A recent study investigating the role of Hla in the virulence of the PVL-negative CA-MRSA strain, ST72, from Korea found that a Hla isogenic mutant did not form any detectable lesions in a mouse skin infection model [54]. Another recent study found that Hla contributed to the virulence of the dominant CA-MRSA strain ST93 from Australia [55]. Hla-deficient ST93 infected mice did not form detectable lesions and the bacterial burden in the skin was significantly lower than wild type infected mice [55]. From these studies it is clear that Hla is an important virulence factor in SSTIs and much of the recent research has convincingly demonstrated that Hla is a major cause of dermonecrosis during SSTIs. Consistent with this, administration of monoclonal antibodies, which neutralised Hla, resulted in reduced lesion size and dermonecrosis in a skin infection model [56].

Phenol-soluble Modulins (PSMs) are small, amphipatic, α-helical peptides. They are grouped into α-type PSMs, which are between 20–25 amino acids in length and β-type PSMs, which are about 44 amino acids long [57,58,59]. In contrast to β-barrel-forming toxins, their ability to lyse cells is thought to be non-specific and receptor-independent [57]. PSMs have an exceptionally high capacity to lyse human neutrophils with neutrophil lysis by PSMα occurring after *S. aureus* has been phagocytosed [60,61]. Abscess size and bacterial burden were significantly decreased in a rabbit skin abscess model when LAC Δ*psmα* was compared to wild type [43]. In another study, Wang R. *et al.* [58], used isogenic mutants of PSM in two CA-MRSA strains, LAC and MW2 (USA400) in a murine skin abscess model. The LAC Δ*psmα* strain had significantly reduced ability to cause skin abscesses when compared to the wild type [58]. *In vitro* assays found that PSMs can effectively lyse human neutrophils and the integrity of the plasma membrane of neutrophils was compromised after only 5 min of exposure to PSMα. The PSM-deletion strains had greatly reduced capacity for lysis [58]. These results suggest that the primary role of PSMs during skin infection is to destroy leukocytes, and thus facilitate *S. aureus* evasion of host immune defence systems.

Taken together the evidence in the literature suggests that dermonecrosis caused by *S. aureus* during SSTIs is almost exclusively caused by secreted toxins. This dermonecrosis causes excessive inflammation, which can lead to further damage of the skin. In addition, the cytolytic effect that these secreted proteins have upon leukocytes acts as an immune evasion mechanism for *S. aureus*, thus facilitating persistence of the infection.

### 4.2. Cell Wall-Anchored Proteins

*S. aureus* expresses an array of cell wall-anchored (CWA) proteins, which are covalently bound to the cell wall peptidoglycan. *S. aureus* can express up to 25 CWA proteins [62]. Many of these can carry out multiple functions and there is functional redundancy between the proteins. All CWA proteins are anchored to the cell wall by transpeptidases known as sortases [63]. Proteins destined for the cell wall contain a C-terminal sorting signal with a conserved LPXTG motif, which is recognised by sortase A. Sortase A cleaves the amide bond between the threonine and glycine, and nucleophilic attack occurs with the pentaglycine chain of peptidoglycan precursor, lipid II, linking the protein to lipid II [63]. This precursor molecule can then be integrated into the cell wall during normal peptidoglycan synthesis. Sortase-deficient mutants, which lack all surface bound CWA proteins, had reduced virulence in murine kidney abscess infection models [64,65]. Wild type bacteria formed abscesses at five days post-challenge, whereas the sortase-deficient strain was unable to cause abscess formation, instead the bacteria were cleared by the animal [64,65]. Similarly, in a skin abscess model, a sortase-deficient mutant had lower bacterial burden in the skin and animals showed a significantly reduced pathology compared to wild type infected mice [66]. These studies clearly indicate that CWA proteins are important during SSTIs. Consistent with this, a rabbit skin infection model, which was used to study the *S. aureus* LAC transcriptome during infection, showed an upregulation of genes encoding CWA proteins including fibronectin and fibrinogen binding proteins and other virulence factors such as proteases and leukocidins 24 h post infection [45].

It is likely that CWA proteins may be particularly important for the initial attachment of bacteria to skin and in the early stages of SSTIs and abscess formation. However, there is a paucity of information regarding the role of individual surface proteins during SSTIs in the literature. Only one study to date has looked at the contribution of multiple individual CWA proteins in a murine skin infection model [66]. The study examined the contribution to pathogenesis of the surface proteins, protein A (SpA), fibronectin-binding proteins (FnBPs), SasF and clumping factor A (ClfA). The mutants used were generated in a number of different strain backgrounds; Newman, SH1000, and LS-1, making direct comparisons between the contributions of each protein difficult. To date, the majority of research into the virulence potential of CWA proteins has been carried out in systemic invasive models of infection. This review draws on the knowledge gained in these models and applies it to SSTIs, providing discussion on a number of CWA proteins, which may be critically important in *S. aureus* SSTI.

#### 4.2.1. Clumping Factor A

ClfA is the major fibrinogen binding protein of *S. aureus* and it binds to the C-terminal region of the fibrinogen γ-chain [67], this can result in platelet aggregation or clumping of bacteria in plasma. ClfA is an important virulence factor of *S. aureus* and its contribution to pathogenesis has been demonstrated in several animal models of infection including endocarditis, arthritis, and sepsis [68,69,70]. In a murine arthritis model, a ClfA-deficient mutant of *S. aureus* showed significantly decreased ability to cause arthritis and also decreased mortality when compared to the wild type Newman strain [68]. In this study, mice were also immunized with recombinantly produced ClfA A domain which conferred some protection from subsequent *S. aureus* infection by the induction of ClfA-specific antibodies [68]. In a murine kidney abscess model of *S. aureus* infection, a ClfA-deficient mutant of Newman had reduced survival in blood and caused decreased bacterial burden in the kidneys compared to the wild type strain. Defects in abscess formation caused by the mutant were not deemed to be significant [65]. These findings suggest that ClfA is primarily involved in pathogen survival and dissemination in blood but not directly involved in forming abscesses in the kidneys.

ClfA also appears to play an important role in skin infection, as mice inoculated with a ClfA-deficient mutant of Newman demonstrated a lower bacterial burden in skin abscesses compared to the wild type strain at Day 2 post-inoculation [66]. In the same study, a Newman strain which had its ClfA gene replaced with a mutated version of ClfA (ClfA PYI), which lacked the ability to bind fibrinogen, was administered, and there was a significant reduction in bacterial burden in the skin abscess, much greater than the reduction seen in ClfA-deficient Newman infected mice. [66]. ClfA protects bacteria from neutrophil phagocytosis through the recruitment of fibrinogen to the bacterial cell surface [71]; this likely inhibits opsonin deposition or shields opsonins from recognition by host phagocyte receptors. In a murine model of septicaemia, fibrinogen-deficient mice are less susceptible to *S. aureus* infection and mice producing a mutant form of fibrinogen lacking the ClfA binding site are more resistant to challenge than wild type mice indicating that the interaction between *S. aureus* and fibrinogen is crucial for facilitation of the infection [72]. Given that fibrinogen is present in damaged skin, it is likely that ClfA binding to this ligand might also be critical for initial attachment of the bacterium in SSTIs. ClfA has been implicated as a potential T-cell antigen [15,73], and this is discussed later in this review.

#### 4.2.2. Clumping Factor B

*S. aureus* attachment to the anterior nares during colonisation is facilitated by the staphylococcal surface adhesin, clumping factor B (ClfB), through high affinity interactions with the cornified envelope. ClfB has been shown to promote nasal colonisation in rodents and humans [74,75,76]. ClfB binds to plasma fibrinogen [77], cytokeratin 10 [78], which is the dominant component of the interior of squamous cells and to loricrin which is the most abundant protein of the cornified envelope of squames [79]. Rates of *S. aureus* nasal colonisation were significantly reduced in loricrin-knockout mice compared to wild type mice, demonstrating that loricrin may be the critical ligand for ClfB *in vivo*, at least in mice [74]. In a murine kidney infection model, a ClfB-deficient mutant of Newman had lower bacterial burden in the kidney; however, no defects in abscess formation were observed using histology methods [65]. This study suggests that ClfB is not involved in abscess formation, at least during systemic infection. As ClfB is known to bind to squames in the nose it is possible that it has a role in the attachment to squames at other locations in the body, and this may be of importance for the initiation of skin infections. The contribution of ClfB to *S. aureus* skin infection and how it interacts with the immune system at this site remains to be elucidated.

#### 4.2.3. Fibronectin-Binding Proteins

Fibronectin-binding proteins (FnBPs) A and B enable *S. aureus* to adhere and invade a range of cell types, including epithelial cells, endothelial cells, fibroblasts, and osteoblasts [80,81,82]. Invasion is facilitated by the host cell fibronectin receptor, integrin α_5_β_1_ [82]. When FnBPA was expressed on the surface of *Lactococcus lactis*, a bacterium that does not normally adhere to fibronectin or endothelium, it conferred the ability to invade endothelial cells [83]. This demonstrates that there is no secondary factor needed to promote the binding of fibronectin and invasion of cells.

The role of FnBPs has been demonstrated in systemic *S. aureus* infection [80,84,85]. FnBPA and FnBPB were found to be crucial to the establishment of sepsis following intravenous challenge, using the strain SH1000 and its *fnbA* and *fnbB* mutants [85]. In this model, all mice infected with the wild type strain died within six days, however, there was a 100% survival rate of mice infected with the mutant strains [85]. They also found that the FnBPA-deficient mutant was unable to form abscesses in the kidneys, which was in contrast to the wild type SH1000, suggesting that FnBPA is indispensible for abscess formation at least in the kidney.

FnBPs have also been shown to play a role in a murine septic arthritis model of *S. aureus* infection with strain LS-1 [80]. An LS-1 mutant of *fnbA* and *fnbB* was not attenuated in its ability to cause arthritis compared to the wild type, however, the mice infected with the mutant had reduced weight loss. This indicates that although FnBPs do not affect the development of local infection within the joint, they significantly contributed to systemic inflammation, characterised by IL-6 production, and ultimately pathology which was characterised by weight loss [80].

A recent study has shown that an FnbAB-deficient mutant of LS-1 had decreased bacterial burden in the abscess on Day 2 post inoculation in a murine skin abscess model [66]. FnBPs have also been demonstrated to play a role in the adherence and invasion of keratinocytes [86], so it is clear from this work that FnBPs may contribute to SSTIs. However, more studies are needed to understand their specific role. Given their propensity to induce systemic inflammation [80] it is possible that they may also contribute to inflammation within the skin, impacting upon abscess development and potentially leading to dermonecrosis.

#### 4.2.4. SasX

SasX is a CWA protein that is believed to have contributed to major epidemics of MRSA infection in hospitals in Asia [87]. The *sasX* gene is encoded by a lysogenic bacteriophage and has spread rapidly among the dominant clones of MRSA in Asia. It has been shown to promote *S. aureus* nasal colonisation in mice by supporting bacterial adherence to desquamated nasal epithelial cells [87]. Immunisation with recombinant SasX or passive transfer of specific antibodies against SasX reduced nasal colonisation [88]. In a skin infection model, mice challenged with SasX-deficient mutants developed smaller abscesses than those infected with the wild type MRSA strain. This suggests SasX contributes to SSTIs, however, it’s specific role remains to be elucidated.

#### 4.2.5. Protein A

Protein A (SpA) is a conserved, multifunctional surface protein of *S. aureus*. Its N-terminus contains five tandemly linked triple-helical bundle domains, which are important for binding to IgG and other ligands, such as TNF receptor 1 (TNFR1) and von Willebrand factor (VWF) [62]. SpA has a number of immunosuppressive traits and is one of *S. aureus*’ most important mechanisms of immune evasion. SpA binds to the Fcγ portion of IgG, resulting in the bacteria becoming coated in IgG bound in the incorrect orientation, leading to decreased recognition by neutrophils and consequently evasion of phagocytosis [89,90]. SpA is also a B cell superantigen that binds to the V_H_3^+^ immunoglobulins on the surface of B cell receptors, causing clonal expansion, which results in apoptosis [91]. This depletion of B cells results in reduced antibody production and the inability to develop robust adaptive immune responses [91]. In contrast to its role in immune evasion SpA can also be proinflammatory. SpA was shown to bind to and stimulate the surface expression of TNFR1 and also its shedding from the cell [92]. TNFR1 binding leads to activation of MAPKs, which results in the expression of IL-8 and other chemoattractant cytokines, ultimately causing neutrophil recruitment. In a murine model of pneumonia, mice infected intranasally with wild type *S. aureus* RN6390 had a significantly higher incidence of pneumonia and bacteraemia than mice infected with the SpA mutant [92]. Mice lacking TNFR1 were also significantly protected from pneumonia, and bacteraemia. Thus, the lack of SpA or TNFR1 expression resulted in reduced bacterial virulence in this pneumonia model.

A murine kidney abscess model was used to determine the contribution of SpA to abscess formation [65]. On Day 5 post-infection, the SpA-deficient Newman infected mice had a significantly reduced number of abscesses compared to the wild type infected mice [65]. Similarly, in an alternative study, mice infected intravenously with SpA-deficient Newman had lower bacterial load and decreased number of kidney abscesses 18 days post inoculation compared to mice challenged with the wild type strain [93]. In this study, immunization with SpA-deficient mutants prior to infection with wild type Newman resulted in a decreased number of abscesses and a reduction in the bacterial burden associated with these abscesses. The sera from mice immunized with SpA-deficient mutants had increased antibody titers towards several staphylococcal antigens, in particular sortase A anchored proteins. This suggests that SpA suppresses the host antibody response during staphylococcal infection. This property of SpA has lead to concerns that protein A could render vaccination futile [4,8].

It is apparent that SpA has a number of functions during infection; however, its role in skin infection has yet to be fully elucidated. When mice were inoculated subcutaneously with a SpA-deficient mutant of Newman, the bacterial burden from skin abscess was significantly lower than abscesses of mice infected with the wild type. The dominant role for SpA during *S. aureus* SSTI is likely immune evasion through its binding of IgG and depletion of B cells, providing the bacteria with time to establish itself and attach to ligands in the skin. It is also likely that SpA is important in driving proinflammatory responses in the skin. The TNFR1 receptor is highly expressed on human keratinocytes [94] and purified SpA was shown to up-regulate the expression of COX-2 and IL-8 by its binding to TNFR1 in human keratinocytes and triggering downstream kinases which result in the activation of NK kappa B and AP-1, ultimately causing skin inflammation [94]. Additionally, the binding of TNFR1 may facilitate binding of *S. aureus* to the skin and in turn the delivery of other virulence factors which may be important in SSTIs.

#### 4.2.6. Iron Regulated Surface Proteins

Iron regulated surface (Isd) proteins are membrane-bound proteins, which contain a near iron transporter (NEAT) motif, which can bind haemoglobin and haem [62]. The proteins IsdA and IsdB are part of the bacterial Isd system, which is a haem uptake system [95]. Iron limitation is an environmental signal often indicative of mammalian host-pathogen interaction. IsdA is a multifunctional *S. aureus* surface protein that has been shown to be important for nasal carriage [96]. IsdA has been shown to play a role in adherence to squames under iron-limited conditions and studies have shown that IsdA promotes adhesion to loricrin, involucrin and K10 [96,97].

In a murine kidney abscess model, mice infected with Newman isogenic mutants of i*sdA* and i*sdB* had reduced bacterial load and reduced abscess formation in the kidneys compared to the wild type infected animals [65]. It is clear that *S. aureus* must require heme-iron scavenging via IsdA and IsdB for expansive growth during infection, so it is not surprising that when these proteins are absent there is a reduction in virulence.

IsdA also functions as a resistance factor against human innate immune defence mechanisms. The presence of IsdA on the surface *S. aureus* causes the bacteria to become more hydrophilic and negatively charged [98]. This contributes to the bacteria’s ability to resist the inhibitory effects of the fatty acids and antimicrobial peptides and allows the organism to colonise human skin more effectively [98,99]. In a rabbit skin infection model transcription levels of *isdB* were increased 24 h post infection [45] suggesting that it may have a role during skin infection. As these proteins bind to ligands found on skin cells, it is likely to play a role in SSTIs, however, this has yet to be characterised.

A number of studies have demonstrated the protective effect of antibodies specific to IsdA and IsdB in murine and macaque models of infection [100,101,102,103]. IsdB was tested by Merck as a vaccine candidate in phase II/III clinical trials. The trials were stopped due to lack of efficacy [4]. This further highlights the current lack of knowledge about what constitutes protection against *S. aureus* infection in humans.

## 5. CWA Proteins as T Cell Antigens

There is a complete lack of insight into how CWA proteins interact with immune pathways, with the exception of SpA, which is known to bind to and stimulate TNFR1 expression [92]. If CWA proteins are to be considered as vaccine antigens, then we need to understand if they are capable of activating T cells. Anti-*S. aureus* vaccines that drive cell-mediated immunity have succeeded in generating antibody-independent protection against systemic infection in mice, provided there is sufficient activation of effector T cell subsets [7,9]. The targeting of individual T cell subsets is now considered an important strategy for next generation anti-*S. aureus* vaccines. To-date, no well-established *S. aureus* T cell epitopes have been identified. It has recently been shown that the majority of adults possess significant levels of circulating antigen-specific memory T cells, presumably indicative of an individual’s prior exposure to *S. aureus* either through commensal colonisation or previous sub-clinical infections [73]. In this study human peripheral blood mononuclear cells (PBMCs) were exposed to a range of *S. aureus* antigens and T cell-specific responses were measured. Extracellular proteins (including secreted proteins and surface bound proteins), elicited greater T cell proliferation and cytokine production than intracellular proteins and thus represent the major T cell antigens of *S. aureus* [73]. Recent work from our group demonstrated that heat killed *S. aureus*, which had been washed to remove all secreted factors in the media, elicits a robust T cell response, suggesting that membrane-bound proteins of *S. aureus* in particular are responsible for activating T cells [15]. Furthermore, we went on to show that ClfA alone can activate T cells at similar levels to that of heat-killed *S. aureus* [15] supporting the idea that CWA proteins in general, and in particular ClfA, may represent important T cell antigens.

A number of studies have already demonstrated the potential of ClfA as a vaccine candidate antigen. Immunisation with ClfA has been shown to confer antibody-mediated immunity in a murine model of *S. aureus* arthritis [68] and a kidney abscess model [104]. Interestingly we have shown that both nasal and oral immunization with targeted nanoparticles, loaded with purified ClfA A domain, led to a Th1 and Th17 cellular immune response in the absence of any humoral response. Nasal immunization with this ClfA-loaded nanoparticle vaccine provided significant protection against systemic *S. aureus* infection. This study demonstrated that a nasal vaccine was capable of protecting against an acute systemic infection and that this protection could be achieved with purely a cellular response. Similarly a vaccine composed of *Candida albicans* agglutinin like sequence 3 protein (Als3p), which has structural and sequence homology with ClfA, was shown to protect mice from staphylococcal septicaemia in a T cell-dependent manner [7]. B cells were neither necessary nor sufficient to provide protection in this model [7]. Interestingly, it has been shown that immunization with ClfA combined with an alum adjuvant failed to confer protection against systemic infection in IL-17A deficient mice, further highlighting the importance of an IL-17 response in protection against Staphylococcal infections [105].

Taken together, these preclinical studies highlight the potential of ClfA as a T cell antigen, and ClfA is currently included as part of a number of multivalent vaccines currently in development. A Pfizer vaccine, currently in phase II clinical trials, is composed of four antigens, including ClfA, MntC and two capsular polysaccharide proteins (CP5 and CP8) [106,107]. The vaccine caused increased antibody production in phase 1 trials and there were no safety concerns, however, no data concerning cellular immunity has been published [106]. A phase I clinical trial was carried out by NovaDigm, using a vaccine composed of the ClfA homologue, Als3p [108]. The vaccine caused a rapid rise in specific antibody titers and induced specific Th1 and Th17 immune enhancement in humans. The vaccine is currently in Phase II clinical trials [107].

ClfA is emerging as a potential potent stimulator of T cells and it is a very promising antigen for vaccine development, however, there has been little or no research carried out on the potential of other CWA proteins to activate T cells. As there are over 20 CWA proteins, studies to determine which proteins elicit high T cell response should be carried out to identify potential proteins for further examination in vaccine studies.

## 6. Conclusions

We know that vaccines targeting T cells are needed to drive a robust immune response against *S. aureus* infection [4,8]. IL-17-producing cells, in particular Th17 and γδ T cells, have been specifically implicated in host defence against *S. aureus* cutaneous infections [14,35]. An ideal vaccine targeted against SSTIs should have three main attributes: (1) induction of antibodies to neutralise toxins which cause dermonecrosis and inflammation; (2) induction of antibodies to neutralise CWA proteins believed to be important in the initial attachment of bacteria to host ligands; and (3) the ability to induce a robust IL-17 response to promote neutrophil recruitment to the site of infection and effective bacterial clearance. CWA proteins, many of which are conserved across strains, could induce antibodies and simultaneously act as T cell activators in a vaccine. This review has highlighted the need for more research to be carried out to understand the role of surface proteins as virulence factors in skin infections and in particular their ability to activate T cells, which is required to inform development of vaccines that can efficiently promote protective immunity to *S*. *aureus* skin infection.
